# High Temperature Induced Glume Closure Resulted in Lower Fertility in Hybrid Rice Seed Production

**DOI:** 10.3389/fpls.2016.01960

**Published:** 2017-01-05

**Authors:** Haoliang Yan, Binglin Zhang, Yunbo Zhang, Xinlan Chen, Hui Xiong, Tsutomu Matsui, Xiaohai Tian

**Affiliations:** ^1^Agricultural College, Yangtze UniversityJingzhou, China; ^2^Hubei Collaborative Innovation Center for Grain IndustryJingzhou, China; ^3^Applied Biological Faculty, Gifu UniversityGifu, Japan

**Keywords:** high temperature, male sterile line, seed production, glume closure, lodicule, rice

## Abstract

Predicted climate changes, in particular, the increased dimension and frequency of heat waves, are expected to affect crop growth in the future seriously. Hybrid rice relies on seed production from male sterile and restorer lines. Experiments were conducted over two consecutive years to compare the high temperature tolerance of parents of different hybrid rice combinations, in terms of fertility rate, flowering pattern, pollination and physiological parameters of the lodicule. Three male sterile lines and a broad compatibility restorer line (as pollen donor and conventional variety as well) were grown to heading stage and then treated with average daily temperatures of 26°C (range 23–30°C), 28°C (25–32°C), and 30°C (26–34°C), respectively, continued for 5–7 days each in a natural light phytotron which simulated the local typical high temperature weather in the field. The results indicated that male sterile lines were more sensitive to high temperature than the restorer line for fertility rate, and the sensitivity varied between varieties. The fertility rate of the restorer line was maintained at about 90% under the high temperature treatments, while it decreased in the male sterile lines by 23.3 and 48.1% at 28 and 30°C, respectively. The fertility rate of the most sensitive line declined by 70%, and the tolerant line declined by 34% at 30°C. Glume closure in the male sterile lines was a major reason for the reduced fertility rate under high temperature, which is closely correlated with carbohydrates content and the vascular bundle pattern in the lodicule. The present study identified a useful trait to select male sterile lines with high temperature tolerance for seed production.

## Introduction

Rice is the staple food for more than half of the world’s population. Rice production in East Asia, Southeast Asia and African countries is important for global food security (Seck et al., 2012). Rice yields in China have been increased rapidly due to the wide application of hybrid rice, which occupies nearly 50% of the total rice planting areas (Cheng et al., 2007; Singh et al., 2015).

Based on the Intergovernmental Panel on Climate Change (IPCC) Fifth Assessment Report (Stocker et al., 2013), the globally averaged temperature in 2012 was 0.85°C higher than in 1880. It is expected that this temperature increment may increase to more than 1.5°C by the end of the 21st century, and the air temperature in China is likely to be 0.3–0.7°C higher in the next two decades than that in 1986–2005 (Shen and Wang, 2013). In the last 117 years, the Yangtze River Basin has had the highest average air temperatures (Tu et al., 1999) and more frequent heat waves than ever before.

Seed production is a critical step in hybrid rice production, in which a male sterile line and a restorer line are used to produce hybrid seeds. Annual hybrid seed production area in China is around 100,000–130,000 ha, average seed yield is 2.4 t/ha and the Yangtze River Basin is the major seed production area (Li et al., 2009; Li and Yuan, 2010; Wang et al., 2011). In recent years, heat or high temperature has become an apparent issue to rice production in Yangtze River Basin, the biggest rice band in China. In 2003, the heat wave happened in late July and up part of August across this area, leading to a big rice yield loss worth up to US$30,700,000 (Tian et al., 2008). Ten years later, a big heat happened again, leading to 60–80% yield reduction in the hybrid rice seed production (Yan et al., 2014, 2015). For hybrid seed production, the parents are sowing in a specific period so that they flower at a period when the extreme temperatures (cold or heat) unlikely happen. However, unpredictable heat shock can still happen, for example, the 2013 growing season. Thus, high temperature has become an important limitation to hybrid rice seed production in China.

The stamen is particularly vulnerable to high temperature (Hasanuzzaman et al., 2013; Mesihovic et al., 2016). For conventional rice varieties, temperatures above 33.7°C can negatively impact the anther dehiscence, which in turn can reduce the number of pollen reaching the stigma and eventually the fertility rate and yield (Satake and Yoshida, 1978; Matsui et al., 2000; Jagadish et al., 2011). However, little is known on how the hybrid rice seed production system responds to high temperature. A number of studies have identified QTLs for heat tolerance at the flowering stage of rice, but genetically how parent plants of hybrid rice adapt to heat stress is lacking (Cao et al., 2003; Xiao et al., 2011; Ye et al., 2012, 2015).

We tested the hypothesis that hybrid rice seed production is more susceptible to high temperature than conventional rice varieties, and genotypic differences for high-temperature tolerance exist among male sterile lines. We treated three male sterile lines and one rice restorer with three different temperatures, and measured fertility rates, flowering patterns, and the physiological parameters of the lodicule to reveal the mechanisms of hybrid rice responding to high temperature.

## Materials and Methods

### Plant Materials

Three male sterile lines—Guangzhan 63S, Y58S, II-32A—and one broad compatibility restorer line 9311 (as a normal pollen donor and a conventional rice variety as well) were selected for this study. The three male sterile lines have been widely used for commercial hybrid seed production; Guangzhan 63S and Y58S have been used in two-line hybrid seed production and II-32A has been used in three-line hybrid seed production.

### Plant Growth and Treatment

A pot experiment was carried out at the experimental farm of Yangtze University (112°31′E, 30°21′N) in 2014 and 2015. The four varieties were planted in four batches of six pots each, and an extra eight batches of the restorer line 9311 were planted in the field (12 m^2^ plot area) to produce enough normal pollen to pollinate the male sterile lines. Planting dates for each line are in Supplementary Tables S1 and S2.

Twenty-day-old seedlings were transplanted into each plastic pot (inner diameter 30 cm, height 30 cm) containing 12.5 kg soil and 8 g N: P: K compound fertilizer (26:10:15). The tillers were cut off leaving only the main stem during the plant culture. The plants were grown to heading stage and then transferred before anthesis to a growth chamber (AGC-MR, Zhejiang Qiushi Environment Co., Ltd, Zhejiang, China) for temperature treatment. The air temperature was dynamically controlled in accordance with the diurnal variation of air temperature simulating local typical heat weather conditions (Supplementary Table S3). Exactly, as the temperature treatment, the average daily temperatures were set to be 26, 28 and 30°C, respectively, with a constant relative humidity of 75%. In each treatment, plants were treated for 5–7 days during flowering and the restorer plants (planted in the field as pollen donor) were transferred to the phytotron next to the male sterile lines for pollination. Hand pollination was carried out to ensure that the spikelet was fully pollinated when the male sterile lines were flowering. The pollen donor restorer plants were transferred from the field to the phytotron every day to ensure an enough pollen supply.

### Flowering Patterns

To clarify the flowering patterns of each line under the various temperature regimes, we counted the spikelets which opened on the panicle each day during anthesis. The number of flowering spikelets in per panicle was counted every 15 to 30 min from 8:00 to 17:00 until all the spikelets were opened on the panicle; newly opened spikelets were counted and marked. At this time, the total number of opened spikelets (spikelets had opened during flowing time) and the number of closure glumes (spikelets without markers, means those spikelets were never opened) were determined for each panicle. The total number of stigma exserted spikelets of each selected panicle was also counted, recognized by stigma traces at the side(s) of the glume. Comparing the flowering periods of the spikelets in male sterile lines with the restorer line 9311, synchronization flowering was defined as the overlap. Otherwise, they were considered as non-synchronization flowering.

The following formulas were used to calculate various parameters:

Percentage of glume closure per panicle = (the number of closure glumes spikelets/the total number of spikelets) × 100%
Percentage of synchronization flowering per panicle = (the number of spikelets synchronized opening/the total number of flowered spikelets) × 100%
Stigma exsertion rate per panicle = (the number of stigmas exserted/the total number of spikelets) × 100%

### Pollinated and Germinated Pollen Numbers on the Stigma

Pollinated pollen numbers were determined as described by Matsui and Kagata (2003). Twenty florets from each line in each treatment were successively collected between 14:00 and 15:00 for 5 days. All samples were stored in an ice box until the stigma was isolated and stained for 10 days with cotton blue. The total numbers of pollen and germinated pollen grains were then counted under a microscope, and the pollen germination rate was calculated.

### Vascular Distribution in Lodicule

Measurements of vascular distribution in lodicule were modified from Wang et al. (1991). In 2014, 20 spikelets were collected from each line in each treatment between 7:00 and 8:00 and fixed in FAA fixative. The lodicule was then detached, rinsed and treated with lactic acid for 12 h until it became transparent. The distribution and number of vascular bundles were recorded using an optical microscope. In 2015, 50 spikelets of each line were random collected from three treatments, measurements were same as in 2014.

### Contents of Soluble Sugar and Starch in Lodicule

Measurements of soluble sugar and starch were modified from Wang et al. (1991). Fifty spikelets of each line from each treatment were sampled between 7:00 and 8:00 during flowering in 2015; the lodicules were isolated and snap frozen in liquid nitrogen. Each sample consisted of 100 pairs of lodicules with three replications. Soluble sugars in the lodicule were extracted with 80% (v/v) ethanol by incubating for 30 min at 80°C and subsequently quantified using the anthrone colorimetric method. The lodicule residue was dried at 60°C and then digested in 4.6 N perchloric acid for starch extraction. The starch in the supernatant was determined using the anthrone colorimetric method.

### Determination of Fertility Rate

Thirty panicles of each line in each treatment were grown to maturity and then harvested to determine the fertility rate (Prasad et al., 2006; Zhao et al., 2010). The fertilized spikelet was determined by its kernel plumpness, both completely and partially filled kernels were considered as fertilized kernels. The fertility rate of each panicle was calculated using the following formula:

Fertility rate = (number of fertilized kernels/total spikelet number) × 100%.

### Statistical Analysis

Statistical analyses were carried out with SAS 9.2. All the data were analyzed by ANOVA to evaluate the main effects of variety and treatment. Duncan tests (*P* < 0.05) were used to detect significant differences between means. Relationships between each index were analyzed by linear regressions.

## Results

### Fertility Rate

Fertility rates in the two-year experiments were shown in **Table 1**. The restorer 9311 was not significantly impacted by high temperatures in term of fertility rate, with an average rate around 90% in 2 years. Fertility rates of the three male sterile lines showed a decreasing pattern along with the increasing temperature (except for the data point of II-32A in 2015), although increasing temperature had larger impact on fertility rate in 2014. Male sterile lines showed dramatically different response to increasing temperature. II-32A has the best tolerance to the higher temperature. Its fertility rate maintain stable or slightly increase when the average temperature increased from 26 to 28°C in 2015. When the temperature was increased to from 26 to 30°C, the fertility rate was decreased by 34.1% in 2 years’ average. Y58S exhibited moderate tolerance to the increasing temperature. When the temperature was increased to from 26 to 30°C, the fertility rate was decreased by 48.4%. On the other hand, Guangzhan 63S exhibited higher sensitivity to increasing temperature. Its fertility rate was decreased by 75.6% when the average temperature increased from 26 to 28°C.

**Table 1:** The fertility rate of each variety with varied temperature (in daily mean temperature) treatment in 2014 and 2015.

Treatment	Fertility rate (%)			
	9311	II-32A	Y58S	Guangzhan 63S
***2014*^a^**				
26°C	92.18 ± 2.22a	30.33 ± 6.84a	45.86 ± 12.79a	12.53 ± 8.21a
28°C	88.41 ± 6.58a	23.47 ± 9.92b	23.24 ± 9.50b	4.32 ± 4.88b
30°C	89.56 ± 3.06a	12.61 ± 7.41c	16.33 ± 9.59c	5.73 ± 4.11b
***2015*^a^**				
26°C	83.93 ± 4.96c	30.63 ± 11.42b	29.65 ± 9.67a	32.24 ± 15.66a
28°C	92.26 ± 1.42a	35.76 ± 5.35a	26.56 ± 9.95b	6.62 ± 3.55b
30°C	87.73 ± 4.10b	37.02 ± 11.99a	22.62 ± 9.58c	7.92 ± 4.48b
***Annual mean*^b^**				
26°C	88.06 ± 5.64b	35.12 ± 4.54a	37.76 ± 13.90a	22.38 ± 15.88a
28°C	90.33 ± 5.10a	35.90 ± 14.07a	35.90 ± 16.01a	5.47 ± 4.38b
30°C	88.64 ± 3.70ab	23.14 ± 10.82b	19.48 ± 10.00b	6.82 ± 4.40b

*^a^Data are means *±* SE. *n* = 25, ^b^*n* = 50, 25 0f 2014 and 25 of 2015. Different letters within a column indicate significant differences at *P* < 0.05 in the same variety.*

### Flowering Patterns

**FIGURE 1:**
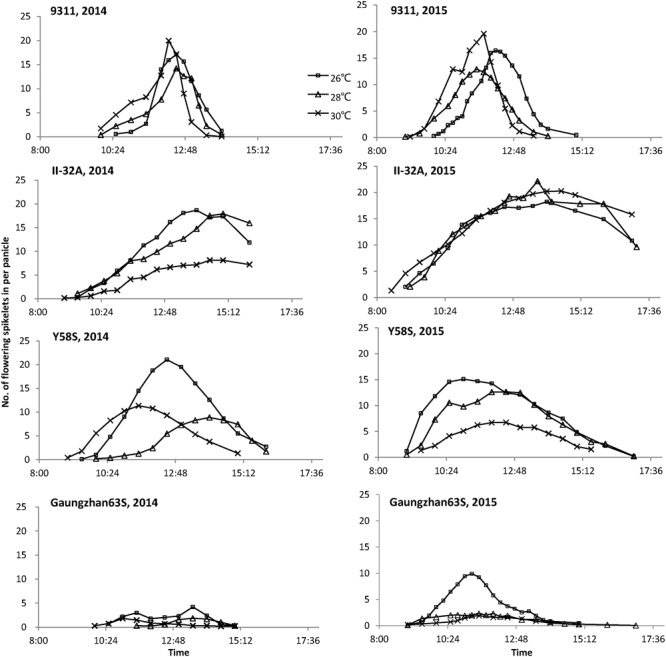
**Daily time course of flowering spikelets of each variety under various temperature treatments.** These figures are based on the data collected from eight panicles of each variety during anthesis in 2014 and 2015.

Flowering time differed between varieties at different temperatures (**Figure 1**). At high temperatures, the restorer 9311 flowered earlier and for a shorter duration than at 26°C, but had little effect on the male sterile line II-32A (except 30°C in 2014). Besides, the fluorescent spikelets of II-32A could be observed from 9:00 to 16:00, indicating a relatively long but scattered flowering pattern. Y58S had different flowering patterns to II-32A, with a longer flowering period at a lower temperature and fewer fluorescent spikelets at higher temperatures. Guangzhan63S showed similar tendencies with Y58S. Besides, Guangzhan63S had lower fluorescent spikelets than other two male sterile lines, especially under 26°C in 2014.

At high temperatures, the shorter flowering duration in the restorer 9311 resulted in fewer florets flowering synchronization with the three male sterile lines (**Table 2**). For instance, at 26°C in 2015, an average of 86.6% spikelets synchronized their flowering time with the male sterile lines but this rate declined to 83.0 and 71.9% at 28 and 30 °C, respectively. II-32A and Guangzhan 63S had significantly lower rates of synchronizing flowering time at higher temperatures. However, compared to that at 26°C, Y58S had a small increase in the rate of synchronizing flowering time at 28°C, due to the earlier blooming time of the restorer line 9311.

**Table 2:** Percentage of synchronization flowering per panicle(against the restorer line 9311), percentage of glume closure per panicle, and stigma exsertation rate per panicle in various male sterile line varieties under varied temperature treatment.

Variety	Treatment	Percentage of synchronization flowering per panicle (%)^a^	Percentage of glume closure per panicle (%)^a^	Stigma exsertation rate per panicle (%)^a^
II-32A	26°C	87.17 ± 0.53a^b^	33.12 ± 2.68a	47.14 ± 2.22b
	28°C	73.58 ± 2.96b	9.61 ± 1.66b	69.98 ± 2.14a
	30°C	68.45 ± 1.54b	14.14 ± 2.80b	65.53 ± 2.82a
Y58S	26°C	76.89 ± 2.45b	7.53 ± 1.69b	79.30 ± 0.76a
	28°C	85.55 ± 1.27a	10.11 ± 0.95b	79.86 ± 0.84a
	30°C	65.76 ± 2.03c	19.37 ± 2.61a	59.18 ± 5.32b
Guangzhan63S	26°C	95.85 ± 1.65a	15.02 ± 3.21b	44.74 ± 5.02a
	28°C	89.64 ± 1.03ab	51.26 ± 3.22a	12.05 ± 2.38b
	30°C	81.38 ± 3.81b	49.81 ± 3.08a	11.79 ± 2.77b

*^a^Eight panicles of each variety were selected randomly during anthesis for data collection in 2015.*

*^b^Data are means ± SE. Different letters within a column indicate significant differences at *P* < 0.05 in the same variety.*

II-32A had the highest percentage of glume closure at 26°C in 2015, which was significantly higher than at higher temperatures. In contrast, Y58S and Guangzhan 63S had a higher rate of glume closure with increasing temperature. Y58S had a remarkable increase in the percentage of glume closure at 30°C, while Guangzhan 63S had a similar pattern even at 28°C.

### Stigma Exsertion Rate

The stigma exsertion rate showed almost a same tendency as the glume closure rate under various temperatures (**Table 2**). The stigma exsertion rate increased with increasing temperature for II-32A but decreased for Y58S and Guangzhan 63S. Guangzhan 63S was more sensitive to high temperature stress than the other lines, having a low stigma exsertion rate at 28°C. In contrast, the stigma exsertion rate for Y58S did not significantly differ at 26°C or 28°C, but dramatically declined at 30°C.

### Pollination Properties

**FIGURE 2:**
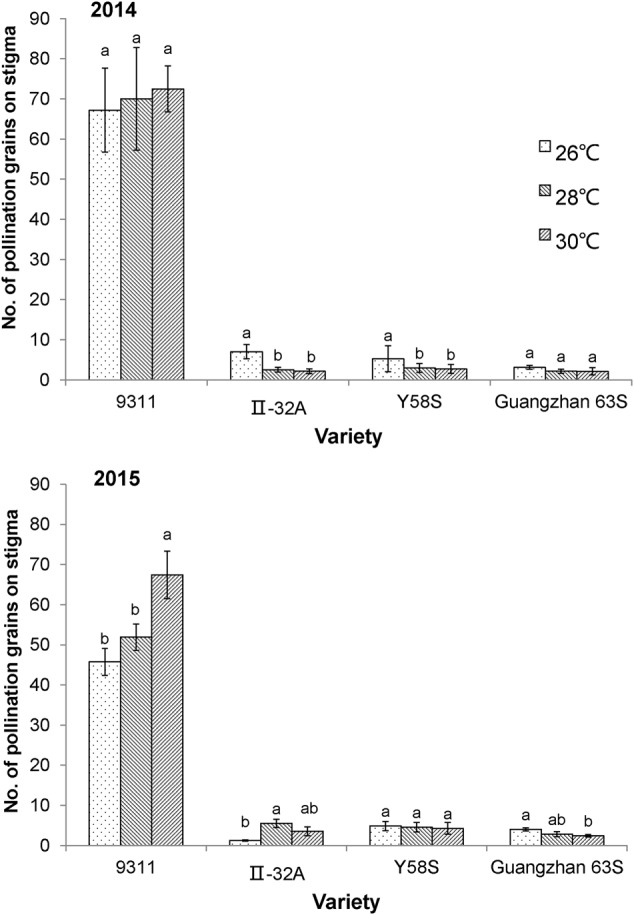
**The number of pollen grains on the stigma in various male sterile line varieties under various temperature treatments.** These graphs are based on data collected in 2014 and 2015. Error bars denote standard errors; different letters represent significant differences at *P* < 0.05 in the same variety.

In 2015, the average number of pollen per stigma on the three male sterile lines was 3.7, which was only 6.7% of the restorer line 9311(9.99 and 4.76% in 2014), indicating that the fertility rate of male sterile lines may be hampered by limited pollen numbers for outcrossing. The number of pollen grains on each stigma increased in the restorer line 9311 at higher temperatures, but the male sterile lines decreased (except for II-32A at 26°C in 2015; **Figure 2**).

**FIGURE 3:**
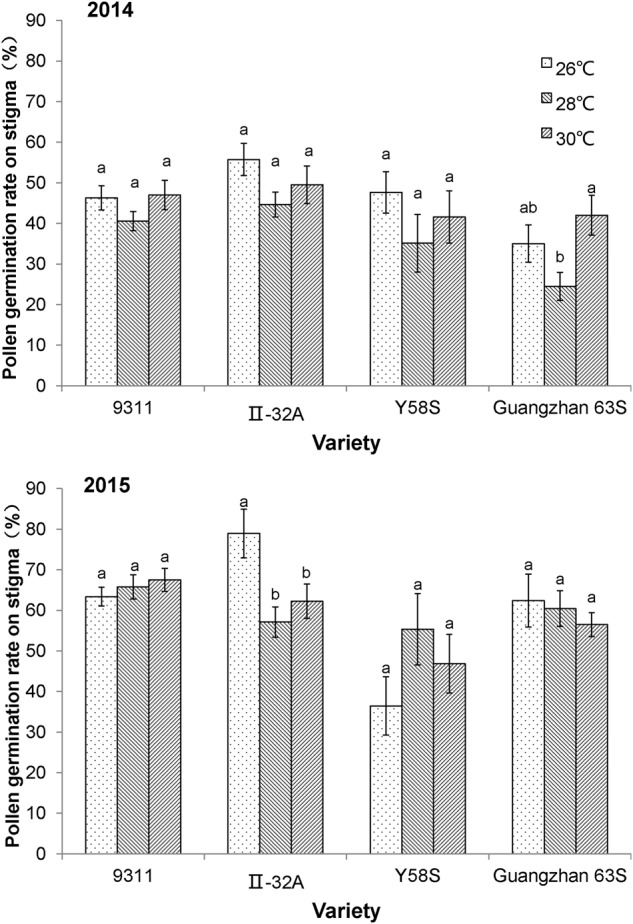
**Pollen germination rate on the stigma of the spikelet in various male sterile line varieties under various treatments.** These graphs are based on data collected in 2014 and 2015. Error bars denote standard errors; different letters represent significant differences at *P* < 0.05 in the same variety.

The percentage of germinated pollen in the restorer line 9311 did not differ in response to temperature in either year, suggesting that both the male and female reproductive organs in the restorer line were not affected by the temperature treatments (**Figure 3**). For the male sterile lines, pollen germination rates for Y58S and Guangzhan 63S did not change with increasing temperatures, while that of II-32A decreased.

### Physiological Characters of Lodicule

**FIGURE 4:**
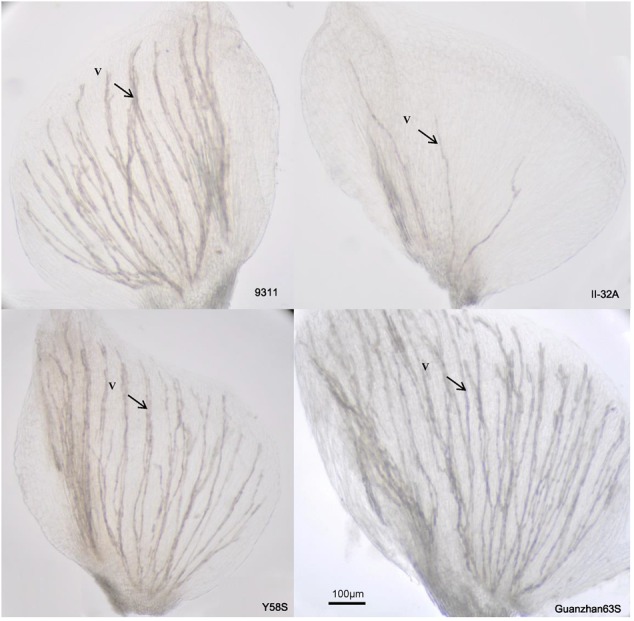
**Photos of vascular distribution patterns in lodicules in various male sterile line varieties.** Photos were taken under an optical microscope after the lodicules were treated with lactic acid for 12 h to become transparent. V: vascular bundles. Photos were taken in 2015.

The number of vascular bundles varied significantly among varieties (**Figure 4**), but not among treatments (**Table 3**). II-32A had the fewest vascular bundles. Y58S and the restore line 9331 had a moderate abundance of vascular bundles, which were four times more than II-32A. Guangzhan 63S had the most vascular bundles.

**Table 3:** The number of vascular bundles in per lodicule.

Treatment	No. of vascular bundles in per lodicule			
	9311	II-32A	Y58S	Guangzhan 63S
***2014*^a^**				
26°C	17.00 ± 0.41a	2.95 ± 0.22a	17.25 ± 0.56a	21.80 ± 0.56a
28°C	16.95 ± 0.47a	3.10 ± 0.20a	18.60 ± 0.53a	22.95 ± 0.46a
30°C	17.30 ± 0.60a	3.00 ± 0.27a	17.10 ± 0.53a	21.70 ± 0.45a
***2015*^b^**				
	17.66 ± 0.43	4.20 ± 0.18	17.34 ± 0.37	21.68 ± 0.35

*^a^Data are means ± SE, *n* = 20.^b^*n* = 50. Different letters within a column indicate significant differences at *P* < 0.05 in the same variety.*

Lodicule starch contents before glume opening did not differ between varieties or among treatments, except for Guangzhan 63S, which significantly decreased at higher temperatures. No significant changes in soluble sugar or starch content in the lodicule were observed in II-32A or Y58S at different temperatures (**Table 4**). The soluble sugar content in the lodicule of 9311 increased with temperature, as did starch content.

**Table 4:** Starch and soluble sugar content in each pair of lodicules in various male sterile line varieties under various treatments.

Variety	Treatment	Starch content in each pair of lodicules (μg)^a^	Soluble sugar content in each pair of lodicules (μg)^a^
9311	26°C	3.96 ± 0.38a^b^	6.28 ± 0.64b
	28°C	3.92 ± 0.92a	7.32 ± 0.81ab
	30°C	4.68 ± 0.67a	8.69 ± 0.64a
II-32A	26°C	2.79 ± 0.32a	7.29 ± 0.74b
	28°C	2.33 ± 0.83a	10.87 ± 0.34a
	30°C	2.59 ± 0.11a	7.94 ± 0.58b
Y58S	26°C	2.91 ± 0.36a	10.96 ± 0.27b
	28°C	2.65 ± 0.78a	13.34 ± 2.19a
	30°C	2.98 ± 0.68a	10.79 ± 0.95b
Guangzhan 63S	26°C	3.89 ± 3.89a	12.93 ± 0.45a
	28°C	3.33 ± 3.33ab	4.76 ± 0.74c
	30°C	2.98 ± 2.98b	7.18 ± 1.06b

*^a^One hundred pairs of lodicules of each line were collected just before flowering to measure the starch and soluble sugar contents in each pair of lodicules; three replications were measured in 2015.*

*^b^Data are means ± SE. Different letters within a column indicate significant differences at *P* < 0.05 in the same variety.*

## Discussion

High temperature can adversely affect crop growth and development, with the reproductive stage generally more sensitive to high temperature than the vegetative stage. The stamen is especially vulnerable to high temperature (Hasanuzzaman et al., 2013; Mesihovic et al., 2016). For conventional rice varieties, temperatures above 33.7°C can impact anther dehiscence and disperse pollen grains, which reduce pollen numbers on the stigma and, eventually, the fertility rate and yield (Satake and Yoshida, 1978; Matsui et al., 2000; Jagadish et al., 2011). Seed production is a critical step in hybrid rice production, where a male sterile line and a restorer line are used to produce hybrid seeds. Success is entirely dependent on cross-pollination. Little information is available on how the hybrid seed production system responds to high temperatures. Our study showed significant reductions in the fertility rate of some male sterile lines when the average daily temperature reached above 28°C or the highest daily temperature reached 32°C compared with 33.7°C observed for conventional rice varieties (Matsui et al., 2000; Jagadish et al., 2011). Our results are consistent with previous reports that the high temperature stress threshold for hybrid rice seed production is around 28°C at the heading stage in the field (Yan et al., 2014, 2015). Our study also confirmed that hybrid rice seed production is more sensitive to high temperature and that the warning temperature for high temperature damage is about 2°C lower in hybrid rice seed production than in conventional varieties.

Cleistogamy is believed to be a protective mechanism against high-temperature damage during anthesis. This phenomenon has been observed in some commercial rice varieties (Li, 1988; Das et al., 2014). Koike et al. (2015) generated a temperature-inducible cleistogamous rice mutant and found that cleistogamy was advantageous to rice pollination and fertilization at high temperatures. However, the hybrid rice seed production system is entirely dependent on cross-pollination between a male sterile line and a restorer line. The cleistogamy mechanism would not be suitable for the male sterile line against high-temperature damage. Instead, glume closure in male sterile lines under high temperature leads to less available pollen for pollination. Our results demonstrated that the fertility rate was significantly associated with the percentage of glume-closed florets (*r* = –0.85^∗∗^, Supplementary Figure S1) and the percentage of exserted stigmas (*r* = 0.97^∗∗^, data not shown). Also, the percentage of exserted stigmas was negatively correlated with the percentage of glume-closed florets (*r* = –0.93^∗∗^, Supplementary Figure S1). Hence, glume closure may be the main reason for reduced fertility rates in hybrid rice seed production at high temperature. For conventional rice, high temperature during anthesis adversely affected anther dehiscence and spreading, which reduced pollination and fertilization as well as fertility rate (Togari and Kashiwakura, 1958; Yoshida et al., 2007). In contrast, the pollination of male sterile lines relies on pollen donation from the male parent (the restorer line). In the present study, the fertility of pollen from restorer line 9311 under various temperature treatments were similar (data not shown), but the average number of pollen per stigma in male sterile lines were 3.7, far less than the lower limits in conventional rice (Togari and Kashiwakura, 1958; Yoshida et al., 2007). Thus, the decreasing fertility rate in male sterile lines was probably due to the fewer stigmas exposed to available pollen from restorer lines, resulting from glume closure at high temperature. Glume closure in response to high temperature should be considered an important trait for heat tolerance. For conventional rice varieties, sensitivity to increasing temperature for glume closure may be a favorable trait for high-temperature tolerance as cleistogamy will reduce the high temperature damage. On the other hand, a lack of sensitivity to increasing temperature for glume closure may be a favorable trait for the male sterile line as this should increase the cross-pollination rate at higher temperatures.

Glume opening/closing in rice is mainly caused by changes in water potential which is regulated by osmotic pressure in lodicules (Wang et al., 1991). The abundance of vascular bundles is favored for the rapid absorption and release of water in lodicules during the opening and closing of the lemma and palea (Wang et al., 1992). In the present study, high temperature reduced soluble sugar and starch contents in lodicules before flowering, both of which were negatively correlated (*P* < 0.001, data not shown) with the glume closure rate, particularly in Guangzhan 63S, which had the largest variation in glume closure rate at high temperature. We also observed that Guangzhan 63S had abundant vascular tissues in lodicules, which is in contrast to II-32A, which had a low abundance of vascular tissues and a broader flowering time. Thus, abundant vascular tissues in lodicules may be a favorable trait for high-temperature tolerance in conventional rice varieties as it could close the glume quickly in response to higher temperature to protect the stamen (Li, 1988; Koike et al., 2015). However, this trait becomes unfavorable for hybrid seed production as glume closure prevents outcrossing pollination. Male sterile lines with less vascular tissue like II-32A should be selected for high-temperature tolerance.

## Conclusion

In summary, seed setting in male sterile lines was more sensitive to high temperature than the conventional rice variety and the fertility rate of some male sterile lines decreased significantly when the average daily temperature was above 28°C, thus the warning high temperature for hybrid rice seed production should be about 2°C lower than the recommended temperature for conventional rice varieties. Meanwhile, the male sterile lines responded diversely to high temperatures, which was directly connected with the trait of glume closure in response under increased temperature. It may be possible to develop high temperature-tolerant male sterile lines with non-glume closure and less vascular bundles under high temperature.

## Author Contributions

XT: responsible for experimental design and organizing; HY: responsible for doing the experiment, data analysis and manuscript preparation; YZ: responsible for assisting in data analysis; BZ: participating in microscopic observation; TM: assisting in experimental design; HX: materials culture; XC: flowering spikelets recording.

## Conflict of Interest Statement

The authors declare that the research was conducted in the absence of any commercial or financial relationships that could be construed as a potential conflict of interest.
