# Supplementary material for: High Temperature Induced Glume Closure Resulted in Lower Fertility in Hybrid Rice Seed Production
Source: Front Plant Sci. 2017 Jan 5;7:1960. doi: 10.3389/fpls.2016.01960 (PMC5214948; doi:10.3389/fpls.2016.01960)
Supplement: Supplementary file 1 [file Table_1.doc]

Supplementary Material

# High temperature induced glume closure resulted in lower fertility in hybrid rice seed production

Haoliang Yan^1,2^, Binglin Zhang^1,2^, Yunbo Zhang^1,2^, Xinlan Chen^1^, Hui Xiong^1^, Tsutomu Matsui^2,3^, Xiaohai Tian^1,2, *^

*** Correspondence:** Xiaohai Tian: xiaohait@sina.com

# Supplementary Figures and Tables

## Supplementary Tables

**Supplementary Table 1.** Sowing batch arrangement for pot experiment for varied varieties.

| Batch | Sowing date(month-date) | | | |
| --- | --- | --- | --- | --- |
|  | Guangzhan 63S | Y58S | II-32A | 9311 |
| Batch 1 | May-24 | May-12 | May-8 | Apr-29 |
| Batch 2 | May-31 | May-19 | May-15 | May-6 |
| Batch 3 | Jun-7 | May-26 | May-22 | May-13 |
| Batch 4 | Jun-14 | Jun-2 | May-29 | May-20 |

**Supplementary Table 2.** Sowing plan of 9311 in the field

| Batch | Sowing date |
| --- | --- |
|  | (month-date) |
| Batch 1 | Apr-20 |
| Batch 2 | Apr-25 |
| Batch 3 | Apr-30 |
| Batch 4 | May-5 |
| Batch 5 | May-10 |
| Batch 6 | May-15 |
| Batch 7 | May-20 |
| Batch 8 | May-25 |

**Supplementary Table 3.** Daily temperature control plan for the phytotron

| Treatment | Temperature setting in different time (°C) | | | | | | | | | | | | | | | | | | | | | | | |
| --- | --- | --- | --- | --- | --- | --- | --- | --- | --- | --- | --- | --- | --- | --- | --- | --- | --- | --- | --- | --- | --- | --- | --- | --- |
|  | 00:00 | 01:00 | 02:00 | 03:00 | 04:00 | 05:00 | 06:00 | 07:00 | 08:00 | 09:00 | 10:00 | 11:00 | 12:00 | 13:00 | 14:00 | 15:00 | 16:00 | 17:00 | 18:00 | 19:00 | 20:00 | 21:00 | 22:00 | 23:00 |
| 26 °C | 26 | 25 | 24 | 24 | 23 | 23 | 23 | 23 | 24 | 25 | 26 | 27 | 28 | 29 | 29 | 30 | 29 | 29 | 28 | 28 | 27 | 26 | 25 | 25 |
| 28 °C | 27 | 26 | 26 | 25 | 25 | 25 | 25 | 26 | 27 | 28 | 29 | 30 | 31 | 31 | 32 | 31 | 31 | 30 | 30 | 29 | 28 | 27 | 27 | 26 |
| 30 °C | 29 | 28 | 28 | 27 | 27 | 27 | 27 | 28 | 29 | 30 | 31 | 32 | 33 | 33 | 34 | 33 | 33 | 32 | 32 | 31 | 30 | 29 | 29 | 26 |

## Supplementary Figures


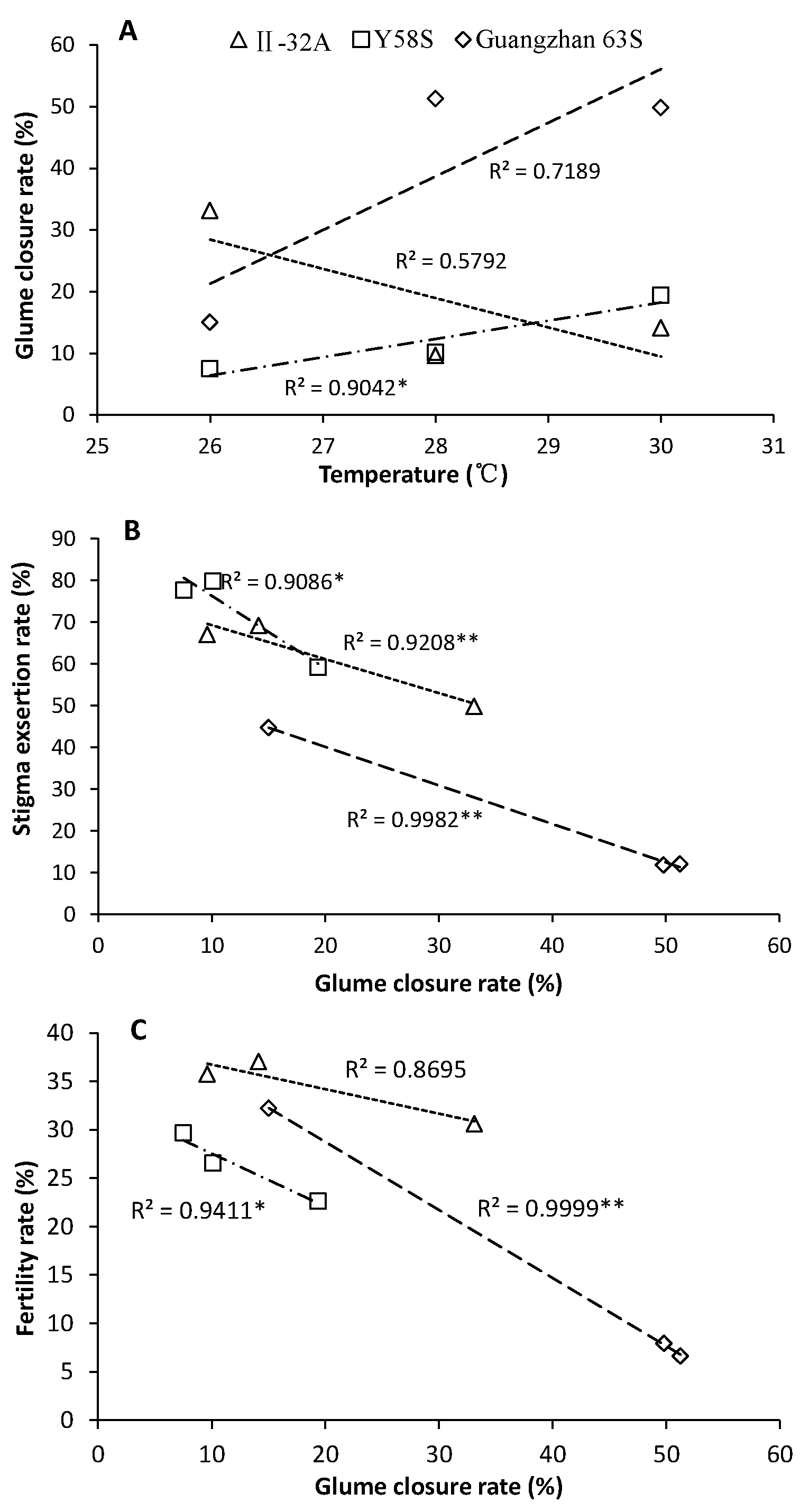


**Supplementary Figure 1.** Correlation between temperature VS glume closure (A), between glume closure VS stigma exsertion rate (B), glume closure VS fertility rare (C). Correlation analyze based on data collected in 2015. * indicate significant relation at P < 0.05, ** indicate significant relation at P < 0.01.
